# How Neoliberalism Is Shaping the Supply of Unhealthy Commodities and What This Means for NCD Prevention

**DOI:** 10.15171/ijhpm.2019.56

**Published:** 2019-07-08

**Authors:** Raphael Lencucha, Anne Marie Thow

**Affiliations:** ^1^School of Physical and Occupational Therapy, Faculty of Medicine, McGill University, Montreal, QC, Canada.; ^2^Menzies Centre for Health Policy, Sydney School of Public Health, University of Sydney, Sydney, NSW, Australia.

**Keywords:** Policy Coherence, Tobacco, Food, Neoliberalism, Governance

## Abstract

Alcohol, tobacco, and unhealthy foods contribute greatly to the global burden of non-communicable disease (NCD). Member states of the World Health Organization (WHO) have recognized the critical need to address these three key risk factors through global action plans and policy recommendations. The 2013-2020 WHO action plan identifies the need to engage economic, agricultural and other relevant sectors to establish comprehensive and coherent policy. To date one of the biggest barriers to action is not so much identifying affective policies, but rather how a comprehensive policy approach to NCD prevention can be established across sectors. Much of the research on policy incoherence across sectors has focused on exposing the strategies used by commercial interests to shape public policy in their favor. Although the influence of commercial interests on government decisions remains an important issue for policy coherence, we argue, that the dominant neoliberal policy paradigm continues to enable the ability of these interests to influence public policy. In this paper, we examine how this dominant paradigm and the way it has been enshrined in institutional mechanisms has given rise to existing systems of governance of product environments, and how these systems create structural barriers to the introduction of meaningful policy action to prevent NCDs by fostering healthy product environments. Work to establish policy coherence across sectors, particularly to ensure a healthy product environment, will require systematic engagement with the assumptions that continue to structure institutions that perpetuate unhealthy product environments.

## Introduction

In 2015, the Zambian government established an incentive program to attract investment in a recently developed multi-facility special economic zone.^1,2^ This program was offered to any company interested in establishing their operations in this zone, including tobacco companies. The decision not to exclude tobacco companies from this program was in direct contravention of the World Health Organization (WHO) Framework Convention on Tobacco Control, which Zambia ratified in 2008. Subsequently, two tobacco processing and manufacturing plants have been established with the expressed aim of targeting the domestic market.^3^ What led to this situation? One explanation is that tobacco interests had influenced or even captured the policy process. Tobacco interests are notorious for exerting influence on policy. But this is only part of the story. Here we argue that ideas, and specifically the neoliberal paradigm, has conditioned the policy environment in a way that promotes the supply of unhealthy commodities.

There is an important need to expand and deepen explanations of the factors that contribute to inconsistencies or incoherence in this policy context. Such insights can help to inform why there is persistent inattention by economic sectors, including trade, commerce, industry and agriculture, to the health consequences of their policies.^4,5^ The most common explanation for policy incoherence pertaining to product-based non-communicable disease (NCD) risk stems from the influence of special interests, particularly unhealthy product producing industries, on policy.^6,7^ Core strategies of corporations that have a direct influence on policy include: defining a strong narrative regarding ‘well-being’ and personal (not government) responsibility, establishing themselves as key stakeholders in consultation regarding the rules of trade and commerce, and influencing the creation of – and use of – knowledge.^4,8^ Scholarship in this area focuses on uncovering the, often nefarious, strategies used by industry to promote unhealthy products and prevent their regulation.^9-11^

Although the influence of private interests on government decisions remains an important issue for policy coherence, and scholarship in this area continues to grow under the new frame of the commercial determinants of health,^12^ we argue, that policy paradigms are a critical but often invisible underpinning of policy (in)coherence, including the ability of private interests to influence public policy. In other words, the accessibility, affordability, and nature of the products in the consumer environment and the ways that companies operate within the market and in relation to government is conditioned by certain conceptions of the proper relationship between government, market and society. Specifically, we illustrate that part of the friction that inhibits healthy product policy regimes is the persistence of the neoliberal paradigm in shaping the relationship between market, state and society. Embedding products in this broader policy context complements scholarship on how the commercial sector influences product regulation by illustrating how policy fosters tobacco, alcohol, and unhealthy foods along the supply chain.^4,13^

Over the past four decades, neoliberal ideas have suffused public policy-making in economic and other sectors. This paradigm has been reified in institutions and created conflict between (1) government policy that continues to foster the production of tobacco, alcohol, and unhealthy foods, and (2) government attempts to foster healthy product environments. In this paper, we examine how neoliberalism became the dominant paradigm, how it has been materialized in institutional mechanisms governing product supply, and how these ways of governing create structural barriers to whole-of-government policy action to prevent NCDs. We focus particularly on the ways that this paradigm has shaped government approaches to product supply in a global market system.

## The Lasting Impact of the Neoliberal Paradigm

Given that neoliberalism can be an elusive construct, we begin with a brief overview of the core tenets of the neoliberal paradigm. To begin, neoliberalism is built upon the “central values” of “individual liberty and freedom as sacrosanct.”^14^ The neoliberal project attempts to translate these basic values into a framework articulating the proper relationship between government, market and society. Ostensibly neoliberalism involves a “restructuring of prevailing ideas, institutions, and material capacities that constitute historical structures of world order”^15^ towards the freedom of individuals to drive the market. Infused in the theoretical origins of neoliberal thought was a societal project aimed at establishing harmony and peace by establishing a market system that transcended particular loyalties and identities. The societal aspiration of the neoliberal project is expressed forcefully by one of its champions Milton Friedman^16^:

“*The great virtue of a free market system is that it does not care what color people are; it does not care what their religion is; it only cares whether they can produce something you want to buy. It is the most effective system we have discovered to enable people who hate one another to deal with one another and help one another.” *

This aspirational undercurrent illustrates that neoliberalism was far more than a set of policy prescriptions but rather, as Peck and colleagues argue, a “hegemonic restructuring ethos.”^17^ Thus, although the policies and practices of neoliberal institutions vary, the underlying paradigm is relatively robust. In this paper, we focus on the paradigmatic (rather than political) aspect of neoliberalism, as an underlying influence on economic policy globally.

Although the underlying rationality of neoliberalism is somewhat clear, where freer markets are thought to foster greater personal freedom thus leading to greater peace and prosperity, the implementation of neoliberal principles into institutional form is better characterized as a “dominant pattern of (incomplete and contradictory) regulatory transformation, and not as a fully coherent system or typological state form.”^17^ However, with the structural adjustment programs (SAPs) beginning in the 1980’s, and the rise of multilateral and bilateral trade and investment agreements, there was a systemic shift to the core program of trade liberalization, privatization, deregulation, property rights among others.^18^ As Peck notes, this program constructs a relationship between government and market with a “orientation to export-oriented, financialized capital; deep antipathies to social collectivities and sociospatial redistribution; and open-ended commitments to market-like governance systems, non-bureaucratic modes of regulation, privatization, and corporate expansion—but these are always, inescapably, forged and revealed in context-specific ways” (p. 104).^17^ The latter point is that the neoliberal program was not uniform in its implementation, however, we can see that the “theoretical template”^14^ of neoliberalism has greatly influenced public policy around the world. One aspect of this neoliberal paradigm that we see translated into economic policy is a reluctance to impose socially oriented protections in favor of a generic rationality of economic growth. In other words, economic growth is given greater policy importance than social goals with the idea that growth leads to enhanced social welfare. This underlying premise leads to a situation where economic policy is often dislocated from social policy.^19^ For example, governments often offer incentives and inducements to companies that produce harmful consumer products with the narrow aim of stimulating economic growth.^20,21^ As Hall^22^ noted in his ground-breaking work on policy paradigms, “when monetarism replaced Keynesianism as the template guiding policy, there was a radical shift in the hierarchy of goals guiding policy, the instruments riled on to effect policy and the settings of those instruments” (p. 285). The instruments of government have thus been heavily influenced by the structuring ethos of neoliberalism.

A highly relevant implication for the management of product environments is the impact this paradigm has had on the governance of agricultural commodities. As noted, specific to this paradigm is the primacy of a narrow economic rationality in approaches to agricultural supply. For example, development plans and trade strategies have typically excluded reference to the health implications of agricultural commodities, at least within the boundaries of licit commodities including food, alcohol, and tobacco. The dominant aim has been to foster free enterprise and competition in global markets. Because these policy approaches are embedded in a global market system, the action or inaction of one country has bearing on the global supply chain. With specific reference to the globalization of the alcohol industry, Jernigan^23^ notes that this process allows large multinational alcohol companies the flexibility to shift global production and distribution to more amendable policy environments. This same flexibility is seen in the operation of tobacco supply whereby companies shift distribution in order to take advantage of lower tariffs or more efficient distribution channels.^24^ This global reality places pressure on governments to compete for investment, which in domains such as labour policy has led to a ‘race to the bottom’ where governments serve to maintain cheap labour or weak labour protections as a form of competitive advantage to attract investment.^25^

One of the clearest examples of a shift in the institutional landscape based on a neoliberal paradigm is the privatization of state-run companies.^18^ This shift is largely attributed to the implementation of SAPs and free trade regimes. The freeing of market structures from state ownership dramatically changed the relationship between state and market, where the role of the state became one of facilitating the ease of global flows of private capital, goods and services. Governments facilitated this process not through deregulation but through what Otero^26^ calls neoregulation. In this case we see that the state has not relinquished itself from regulating agricultural supply but rather has restructured its involvement in accordance with the governance (ie, public-private management) of supply chains. One example of this shift is the rise in contractual arrangements between agro-based companies and smallholder farmers in the neoliberal era.^27^ These arrangements shift the management of production from government to private entities who provide credit, agricultural inputs such as seed, fertilizer, and equipment on loan, and transportation to market. Although this is not uniformly true across agricultural commodities, this shift is relatively consistent for cash crops such as tobacco, oilseeds, and other commodities that have health implications for consumers. For example, governments in tobacco-producing countries have largely been replaced by leaf-buying companies in managing the supply chain, whereby these companies provide extension services, inputs (eg, seed, fertilizer) on loan, cash loans, transportation to market, and other services to tobacco farmers.^28-32^

This privatization of agricultural supply chains is seen by economic actors as a strategy to increase the quality and quantity of agricultural outputs while ensuring markets for agricultural commodities. However, it has implications for the capacity and willingness of government to engage in supply chain management. In particular, governments’ have a weakened, or at least a more indirect, ability to shape the supply of these commodities. Research into tobacco production suggests that farmers continue to grow tobacco largely because companies provide access to inputs such as seed and fertilizer as well as cash loans and access to markets.^33-35^ Similarly, supply chains for minimally processed healthy foods such as fruit and vegetables are often characterised by significant losses, due to a systemic lack of public investment in agriculture.^36^ In contrast supply chains for highly processed vegetables, such as potato chips are highly developed due to downstream industry investment. This dynamic is characteristic of the neoliberal paradigm representing a general “decline in central government’s ability to steer society.”^37^ The force of law is used to protect private individual and corporate rights while ensuring that the international movement of goods, services and capital is freed, and that this freedom is protected. Another way that these impacts are being perpetuated is the embedding of companies in regulatory bodies. For example, in Malawi, tobacco leaf prices are established by institutions such as the Tobacco Control Commission and the Agricultural Research Extension Trust, both of which have industry representation on their decision-making bodies.^38^ All of these shifts in the relationship between government and market are consistent with an economic paradigm that views the role of government as facilitator of freer market activity.

## The Institutionalization of Neoliberalism

Neoliberal ideology finds its force in the establishment of institutions that orient policy towards the principles outlined above. Ostrom characterizes institutions as the rules, norms and strategies that shape action taken by government.^39,40^ In a nuanced analysis of the ‘old’ and ‘new’ instruments of government, Jordan and colleagues^37^ suggest that ‘old’ forms of government characterized by ‘strong’ government action, whereby government sets the agenda and establishes mechanisms of command-and-control, still exist but have given way to ‘new’ instruments. These new instruments are characterized by varied levels of involvement with and control by non-governmental entities. This shift in the instruments used by government is exemplified by the pervasive use voluntary codes and standards in meeting health-based targets in the reduction of sodium and other unhealthy ingredients in foods.^41,42^

There are different mechanisms of reification that have structured governments’ relationship to agricultural production, commodity processing and manufacturing and the management of consumer demand. The embedding and reification of neoliberal ideas is in part the result of diffusion through powerful global institutions, as evidenced by the dominance and global impact of the SAPs driven by the International Monetary Fund and the World Bank^15,43^ and the international trade and investment regimes that have the ability to discipline governments for policies that did not support the opening of markets to competition.^44,45^ The domestication of this paradigm can be found in national development plans, export development strategies, and the ministerial mandates of economic sectors of government.

Although we have emphasized the structuring of the neoliberal paradigm in the policy and institutional landscape, alone this perspective is overly deterministic. To temper this structural dimension, it is important to turn to ideas, discourses and their relationship to policy change.^46,47^ The ‘new institutionalism’ provides important insights into how institutions shape the preferences and behavior of decision-makers while articulating the dynamic capacity of agents to shape institutions. Two of the central assertions of new institutionalism is that “preferences or interests expressed in action should not be conflated with ‘true preferences” and that “institutional configurations may privilege particular sets of interests.”^46^ In other words, the perpetuation of policy paradigms is at least in part driven by institutional legacies. These legacies are reflected in ‘real’ institutions. These institutions are at one level structuring, whereby government decision-makers enter into pre-existing institutions with particular historical legacies, mandates, bureaucratic arrangements, etc. At the same time government decision-makers, civil society and non- and inter-governmental actors have power to shape these institutions to modify mandates, reconfigure bureaucratic arrangements and create new rules. The insight that institutions do not always reflect the preferences of individuals operating within them recognizes that although institutions shape action, contestation exists within institutions. From a practical perspective, this insight has implications for how one approaches efforts to establish policy coherence. It is common to conflate problematic policies with the preferences of policy-makers. However, institutional structures can preference certain actor perspectives, such that policy-makers are differentially exposed to input in policy-making, which aligns with the dominant paradigm. Industry actors are seen as major legitimate stakeholders by economic policy actors because within a neoliberal paradigm the private sector is critical to their mandate to support economic growth and employment. For example, alcohol policy in Lesotho, Malawi, Uganda, and Botswana was heavily influenced by industry actors. What this meant for public health is that the policies selectively adopted international recommendations and minimized a public health approach to alcohol problems, while emphasising the economic benefits from trade in alcohol.^48^

What we are arguing is that the neoliberal paradigm is a critical driver of the tension that arises when the health sector attempts to intervene in the supply of unhealthy products governed by economic sectors. For example, economic norms aligned with the neoliberal paradigm have suffused the discourse and institutions in tobacco growing countries creating recurrent patterns of argumentation against tobacco control that transcend local contexts.^49,50^ In countries like Zambia, Malawi and Kenya, the development plans have given preference to mandates of economic ministries, and include efforts to defend tobacco production even though evidence contradicts its economic viability.^31,32^ Similarly, in South Africa, the dominant economic policy paradigm addresses food as a commodity – pivotal for employment and rural development – with minimal consideration of nutrition implications of the food environment.^51^

However, as Schmitt argues, institutions are dynamic sites of contestation (not static objects), in which individuals have the capacity to participate in discursive processes that shape both the ideational basis of the rules, norms and strategies that constitute the institutional environment.^52,53^ There are examples of recent shifts in national policy regarding unhealthy agricultural commodities such as tobacco, driven in part by concerted efforts of international institutions such as the United Nations Development Programme to integrate health with economic development.^54^ For example, Malawi’s most recent national export and development strategies emphasize the need to move away from tobacco production into other agriculture commodities such as oil seeds. Although these policies use economic framing, there is evidence that the health discourse is beginning to shape discourses in the economic sector or at least the economic discourses are no longer isolated from the messages of health advocates confronting a narrow economic rationality.^55-58^ This contestation is exemplified at the intersection of trade and tobacco control whereby tobacco control proponents, drawing from government commitments to the Framework Convention on Tobacco Control, have worked to infuse health goals in the logic of economic sectors.^49,57,59^

## Implications for Policy Action

Our aim in articulating the paradigmatic tensions that exist within government, the institutions that are born from these different paradigms, and the ways that these institutions approach the management of product environments is to, in part, sensitize health advocates to the issues facing colleagues in the economic sector. We conclude by discussing how this understanding can shape approaches to policy coherence for healthy product environments.

First, the influence of health-harming industries on public policy is real and deeply problematic for public health. As illustrated above, the relationship between policy-makers and industry is often far too close and conflicts of interest, in many cases outright corruption, abound. It is clear that institutions must be reoriented to protect public policy from private interests through enforceable rules of engagement if health protection is going to be a whole-of-government initiative. One prominent example of such an initiative is the Guidelines for the Implementation of Article 5.3 of the Framework Convention on Tobacco Control, which establishes principles and recommendations to guide interactions between government and the tobacco industry.^60^ The ultimate aim is to educate all sectors of government about the harmful impacts of tobacco industry practices, reduce or eliminate industry involvement in policy processes and where this is not possible, ensure transparency when involvement occurs. Although the implementation of Article 5.3 has faced challenges, it is one example of how norms can infuse institutions to ensure government officials distance themselves from industries that produce health-harming goods.^61^

Second, there are practical implications of attending to the conditions that foster policy incoherence. The context of cooperation between sectors is often strained. This strained relationship is often fueled by mistrust and competing viewpoints on an issue.^62^ Although it is commonly understood that different viewpoints or frames can create tension and distance between sectors, few studies have articulated the paradigmatic assumptions that structure such viewpoints. The perspective we are advocating for in this article compliments work that interrogates these paradigmatic assumptions underlying tensions between trade and health^62,63^ by identifying how the neoliberal paradigm has structured the institutional environment in economic, agricultural and other sectors that shapes the supply of unhealthy products. The perspective we are advancing here is that by understanding this paradigmatic and institutional context health advocates can be more sympathetic to policy-makers in these sectors. Contrary to the assumption that policy-makers in these sectors are willfully aligned with commercial interests, this perspective illustrates that government officials are often bound up within historically structured patterns of thinking and doing that foreground economic interests over health considerations. For example, analysis of the intersectoral governing body on tobacco established in the Philippines revealed that the representatives from the economic sector of government viewed the tobacco industry as a legitimate stakeholder based on the perspective that they were a legal enterprise contributing to the economic goals of the country. These officials sought to ‘balance’ economic and social goals in the operations of this intersectoral body.^64^ Not surprisingly, this perspective created animosity between economic and health sector representatives. Interestingly, Lang^65^ argues that the dominance of the neoliberal paradigm has even shaped the notion of ‘balance’ between economic and social goals in a way that perpetuates the implicit dominance of a narrow economic rationality over a socially oriented economic policy. All this to illustrate that neoliberal ideas are often not explicated or interrogated when it comes to efforts to establish policy coherence.

Third, and stemming from the point above, we hope that this perspective widens the scope for creative solutions to the problem of policy incoherence. For example, rather than simply focusing on the regulation of unhealthy products, there can be a shift towards achieving healthy product environments by engaging with sectors such as agribusiness who are charged with ensuring economic growth and facilitating the economic livelihoods of farmers.^66,67^ Addressing supply side issues serves to break down silos that indirectly or directly provide industries that produce unhealthy products an opportunity to engage with policy-makers. In other words, the supply side issues are often neglected by health advocates and because of this neglect, the supply side is driven by relationships between industry and government supported by conditions that encourage such relationships in the spirit of economic development. By engaging with decision-makers involved in supply side issues health advocates have an opportunity to create dialogue across paradigms and interrogate and debate the merits of these paradigmatic foundations with those who are operating to foster product supply.

## Conclusion

The work articulating how interests have shaped public policy, often in contradiction to the goals of health ministries, has provided important insights into barriers to policy coherence. Analysing the commercial determinants of health provides insights into why public policy often makes the healthy choice the difficult one for consumers. The question that has received less attention is what underlying conditions have shaped a policy environment that is conducive to the influence of commercial interests. Contrary to public health concerns regarding the influence of private commercial interests in the institutional environment where policy decisions are made and enacted, economic sector actors often see this involvement as part of the mandate of stakeholder engagement. This disconnect across sectors cannot simply be explained by corporate lobbying or nefarious relationships between private and public entities. What we have attempted to illustrate is that these relationships are a logical extension of the neoliberal paradigm that has for four decades dominated the imagination of the policy sphere serving to structure the institutional landscape in countries around the world. Work to establish policy coherence across sectors, particularly to ensure a healthy product environment will require systematic engagement with the assumptions that continue to structure institutions across sectors.

## Ethical issues

Not applicable.

## Competing interests

Authors declare that they have no competing interests.

## Authors’ contributions

RL developed the idea for the manuscript in consultation with AMT and wrote the first incomplete draft. AMT contributed significantly to the development of the manuscript. RL and AMT contributed to each iteration of the manuscript and agree to the final content.

## Authors’ affiliations

^1^School of Physical and Occupational Therapy, Faculty of Medicine, McGill University, Montreal, QC, Canada. ^2^Menzies Centre for Health Policy, Sydney School of Public Health, University of Sydney, Sydney, NSW, Australia.
